# Attachment of *Agrobacterium* to plant surfaces

**DOI:** 10.3389/fpls.2014.00252

**Published:** 2014-06-05

**Authors:** Ann G. Matthysse

**Affiliations:** Department of Biology, University of North CarolinaChapel Hill, NC, USA

**Keywords:** *Agrobacterium*, attachment, adhesion, exopolysaccharides, bacterial binding

## Abstract

*Agrobacterium tumefaciens* binds to the surfaces of inanimate objects, plants, and fungi. These bacteria are excellent colonizers of root surfaces. In addition, they also bind to soil particles and to the surface of artificial or man-made substances, such as polyesters and plastics. The mechanisms of attachment to these different surfaces have not been completely elucidated. At least two types of binding have been described unipolarpolysaccharide-dependent polar attachment and unipolar polysaccharide-independent attachment (both polar and lateral). The genes encoding the enzymes for the production of the former are located on the circular chromosome, while the genes involved in the latter have not been identified. The expression of both of these types of attachment is regulated in response to environmental signals. However, the signals to which they respond differ so that the two types of attachment are not necessarily expressed coordinately.

## INTRODUCTION

Most terrestrial bacteria are found living on surfaces. *Agrobacterium tumefaciens* lives in the upper layers of the soil and in the rhizosphere. These bacteria can bind to a variety of inanimate surfaces including quartz sand, glass, plastic, polyester, and cellulose (Tomlinson and Fuqua, 2009). Considering the range of substrates to which the bacteria are able to bind, the bacteria presumably can also bind to particles in the soil. In addition, *A. tumefaciens* binds to the surface of plants, particularly to roots and root hairs, and to the surface of fungi (Matthysse et al., 1978; Bundock et al., 1995; Matthysse, 1996; Piers et al., 1996). Roots release a number of organic compounds into the soil including dicarboxylic acids, amino acids, and sugars (Lugtenberg et al., 1999). Thus, the colonization of the root surface may be advantageous for *A. tumefaciens*. That binding to roots promotes bacterial growth is illustrated by the interaction of two isogenic strains of *E. coli* differing only in adhesin genes which can and cannot bind to alfalfa sprouts (Jeter and Matthysse, 2005). When the strains are inoculated individually with the sprouts only the strain which can bind grows. In addition, when the strains are inoculated together, once again only the strain which can bind grows. Thus, the binding of one strain did not promote the binding or growth of the other strain. The experiment suggests that binding to the root would confer a considerable advantage over simple presence in the rhizosphere. Binding to the root also results in the formation of a biofilm (Ramey et al., 2004). Many studies have shown that bacteria in biofilms, such as those on the root epidermis, are protected from toxic compounds including antibiotics and from predation by protists (Ramirez and Alexander, 1980; Stewart and Costerton, 2001; Danhorn and Fuqua, 2007).

## EARLY STUDIES OF THE ATTACHMENT OF *A. tumefaciens* TO PLANT CELLS

The importance of bacterial attachment to the plant surface was first recognized by Lippincott and Lippincott (1969). They showed that prior exposure of the plant wound site to avirulent *A. tumefaciens* resulted in inhibition of tumor formation by virulent bacteria and that the mathematics of the inhibition fit a one-particle dose–response curve suggesting that the avirulent bacteria were occupying sites and making them unavailable to the virulent bacteria. Additional studies of attachment of *A. tumefaciens* to plant cells and wound sites were carried out in the next 20 years. The techniques generally used in these early studies of attachment rely on indirect measurements of bacterial adhesion: competition between various bacterial strains as seen in the experiment described above, removal of bacteria from sites by washing (Lippincott and Lippincott, 1967), and inhibition of tumor formation by treatment of the wound site or the bacteria with surface extracts of the bacteria or plant cells prior to inoculation of the bacteria into the wound site (Whatley et al., 1976; Lippincott et al., 1977; Neff et al., 1987; Wagner and Matthysse, 1992). The first method requires that there be a limited number of discrete attachment sites where bacterial binding can initiate tumors so that the avirulent strain can occupy these sites and block binding of virulent bacteria. It has the advantage that only binding to sites which result in tumor formation is measured. The second method only produces results if the bacteria are bound reversibly. The third method depends on the extracts being tested having no other effects on the plant or bacterium in addition to their effects on the binding site. These experiments were carried out when there was little information on plant defense responses to bacteria and many of them are difficult to interpret due to possible stimulation of plant defense responses by the extracts which could then inhibit tumor formation without having any significant effect on bacterial binding. Extracts which were shown to inhibit tumor formation include pectin (Lippincott et al., 1977; Neff et al., 1987), bacterial lipopolysaccharides (LPS) (Whatley et al., 1976), and plant cell wall proteins (Gurlitz et al., 1987; Wagner and Matthysse, 1992). Reviews of experiments prior to 1986 concerning attachment of *A. tumefaciens* to plant cells have been published by Lippincott and Lippincott (1975) and Matthysse (1986).

Direct observations of bacterial binding to plant cells have been made using plant tissue culture cells and seedling roots of a variety of plants including *Arabidopsis thaliana*, tomato, tobacco, and carrot. Microscopic studies have the advantage that the site and orientation of bacterial attachment can be observed. Their major disadvantage is that large numbers of bacteria are usually required. Bacterial attachment can also be measured using radioactive bacteria or by washing the tissue and determining the number of bacteria bound (retained) using viable cell counts. Washing the tissue has the advantage that reversible and irreversible binding can be distinguished (Neff and Binns, 1985). These methods allow detection of small numbers of bacteria but they may remove (and thus fail to detect) bacteria which are loosely bound to the plant tissue.

## POLAR ATTACHMENT MEDIATED BY THE UNIPOLAR POLYSACCHARIDE (UPP)

**FIGURE 1:**
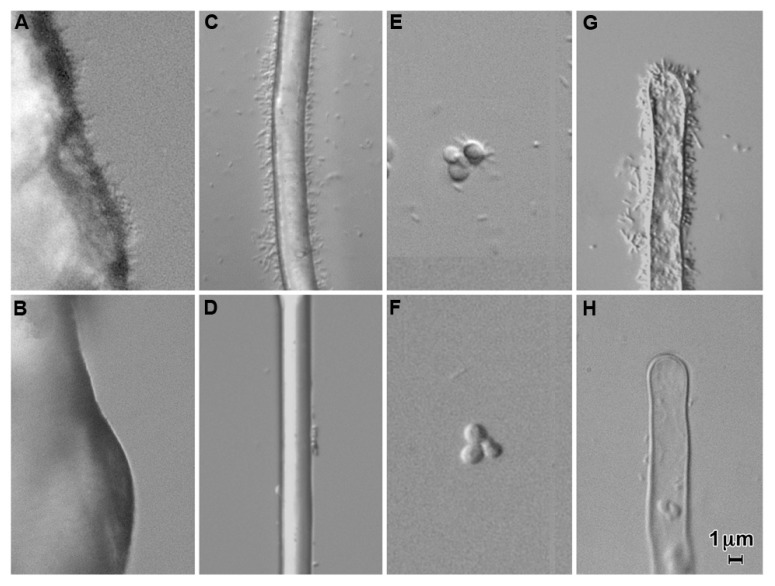
**Attachment of *Agrobacterium tumefaciens* strain C58 (A,C,E,G) and a UPP-deletion mutant of C58 (B,D,F,H) to quartz sand (A,B), polyester thread (C,D), yeast (*Saccharomyces cerevisiae*; C,F), and tomato root hairs **(G,H)** in a 1/10 dilution of MS medium containing a 1/20 dilution of AB minimal medium**. Note the copious attachment of wild-type cells and the large decrease in attachment in a UPP deletion mutant. Approximately 10^6^ bacteria per ml were incubated with the substrate for 24 h.

Visually, the most prominent type of attachment of *A. tumefaciens* to surfaces under a variety of conditions is polar binding of the bacteria (for example, see **Figure 1**). On root hairs or polyester threads, polar attachment of bacteria gives the appearance of a bottlebrush. This binding occurs early in the interaction of the bacteria with both biological (plant and fungal) and non-biological surfaces (Li et al., 2012). Polar attachment of *A. tumefaciens* is mediated by the unipolar polysaccharide (UPP; Tomlinson and Fuqua, 2009). This extracellular polysaccharide was first described in *Rhizobium leguminosarum* where it mediates polar attachment to root hairs (Laus et al., 2006). The *R. leguminosarum* UPP has been shown to be composed largely of mannose and glucose (Laus et al., 2006; Williams et al., 2008). Lectins from the plants nodulated by this bacterium, pea and vetch, bind the polysaccharide. *R. leguminosarum* mutants which are unable to make the UPP are deficient in binding to root hairs under acidic conditions (pH 5.6) but not under more alkaline conditions (pH 7.2) in the presence of calcium ions (Laus et al., 2006; Downie, 2010). *A. tumefaciens* makes a similar polysaccharide localized to one pole of the cell (Tomlinson and Fuqua, 2009). The genes required for its synthesis are located in two adjacent operons (*Atu1235–Atu1239*) in *A. tumefaciens* strain C58. Deletion of these genes results in mutant bacteria which fail to show prominent polar binding to inanimate surfaces, fungi, and plants (**Figure 1**). The formation of the UPP is required for biofilm formation on a wide variety of surfaces (Danhorn and Fuqua, 2007).

The UPP reacts with wheat germ agglutinin (WGA), a lectin which binds to *N*-acetyl-glucosamine (Tomlinson and Fuqua, 2009; Xu et al., 2013). Fluorescent WGA has been used to visualize the presence of the UPP in bacteria growing under various circumstances. Studies using fluorescent WGA have shown that the UPP is rarely made by planktonic bacteria (Li et al., 2012). Shortly after the bacteria come into contact with a surface, UPP is visible at the attached pole (Tomlinson and Fuqua, 2009; Barnhart et al., 2013; Xu et al., 2013). How the bacteria detect the presence of a surface and how this triggers the elaboration of the UPP is not known.

Attachment of bacteria to surfaces mediated by the UPP appears to be irreversible. Bound bacteria are retained after washing of the substrate to which the bacteria are bound (Tomlinson and Fuqua, 2009; Barnhart et al., 2013). In particular, the washing required for the detection of the UPP by fluorescent WGA does not appear to remove the bacteria.

Several genes and environmental conditions involved in the regulation of the production of UPP have been identified. These include concentrations of phosphate (Xu et al., 2012) and calcium (Matthysse, manuscript in preparation) in the environment and regulation via the intracellular, signal molecule cyclic-di-guanylic acid (c-di-GMP) in response to unidentified signals (Xu et al., 2013). The increased binding and biofilm formation seen with phosphorus limitation is dependent on the presence of functional UPP genes in the bacteria. Overexpression of the regulator involved in the uptake of phosphorous, *phoB*, increases the amount of UPP present and thus bacterial surface binding (Xu et al., 2012). Increased calcium ion concentrations (3 mM or greater) cause a reduction in UPP and a consequent decrease in polar bacteria binding (Matthysse, manuscript in preparation). The mechanism of this effect is unknown. The *exoR* gene involved in the regulation of succinoglycan synthesis and flagellar gene expression is also involved in the regulation of biofilm formation (Tomlinson et al., 2010). A deletion of *exoR* results in decreased biofilm formation on roots but individually bound bacteria are still seen. ExoR mutants retain virulence. c-di-GMP also plays a role in the regulation of the production of the UPP (Xu et al., 2013). Constitutive expression of *pleD*, a diguanylate cyclase also called *celR*, results in the synthesis of UPP not just at the pole of the cell but distributed all over the bacterial surface. Deletions of a gene *visR* required for motility result in increased biofilm formation and increased the production of the UPP. VisR was shown to inhibit the expression of the diguanylate cyclase genes *dcgA* and *dcgB* and thus a deletion of *visR* should increase their activity. Mutations in a guanylate phosphodiesterase (*Atu3495*) resulted in higher levels of c-di-GMP and of the UPP (Xu et al., 2013). When VisR is expressed, the cells are motile and the synthesis of UPP is inhibited due to the lack of synthesis of c-di-GMP by DcgA and DcgB. Thus the regulation of the elaboration of the UPP is complex and is integrated with pathways in the bacterium controlling motility (*visR* and *exoR*), regulation of other exopolysaccharides (*exoR* and *pleD* aka *celR*), and phosphate uptake (*phoB*).

Binding to surfaces involving the UPP does not require the presence of the Ti plasmid and strains lacking pTi show binding indistinguishable from that of virulent strains (Tomlinson and Fuqua, 2009). None of the regulatory pathways involved in the control of UPP synthesis are known to be influenced by genes located on pTi. A UPP deletion mutant retains virulence on all plants tested including *Kalanchoe daigremontiana*, potato, and tomato (Tomlinson and Fuqua, 2009). Thus it seems likely that there is a second mechanism of attachment of the bacteria to the plant surface which is involved in the transfer of the T DNA.

## UPP-INDEPENDENT ATTACHMENT

**FIGURE 2:**
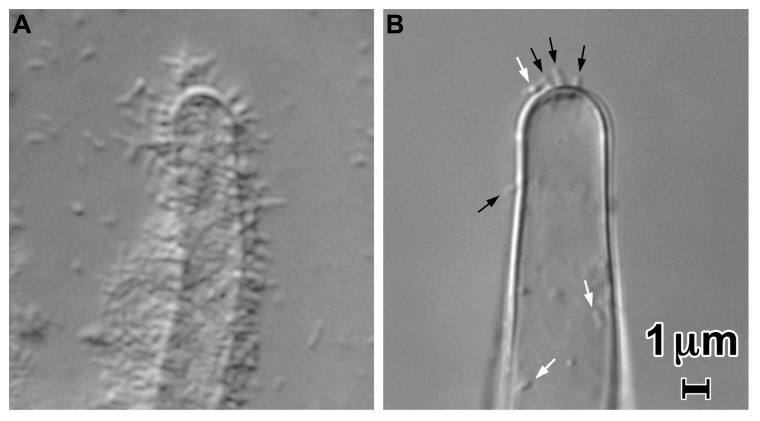
**Attachment of *A. tumefaciens* strain C58 to tomato root hairs in a 1/10 dilution of MS medium containing a 1/20 dilution of AB minimal medium (A) and in 60 mM CaCl_2_, 7 mM sodium acetate, and 247 mM mannitol pH 5.8 (B)**. Note the large decrease in attachment in the presence of the CaCl_2_-containing medium Laterally attached bacteria are visible at the white arrows in **(B)**; polarly attached bacteria are indicated by the black arrows. Approximately 10^6^ bacteria per ml were incubated with cut tomato roots for 24 h.

Although the UPP mediates the visually and numerically prominent polar binding of *A. tumefaciens* to surfaces, it is not required for virulence (Tomlinson and Fuqua, 2009). In a UPP deletion mutant or under conditions in which the UPP is not made, bacterial binding to the surface of plants can still be observed (**Figure 2**). This binding involves very few bacteria compared to that mediated by the UPP. It may require the presence of the Ti plasmid. Attachment of *A. tumefaciens* strain C58 to carrot suspension cells incubated in Murashige and Skoog medium (MS) was observed to be dependent on the presence of the Ti plasmid (Matthysse et al., 1978) as was bacterial attachment to protoplasts in a medium containing 60 mM CaCl_2_, 7 mM sodium acetate, and 247 mM mannitol pH 5.8 (Aguilar et al., 2011). The number of bacteria observed to be attached was low in both of these experiments. In MS medium, bacterial binding to tissue culture cells and root hairs was both polar and lateral. In 60 mM CaCl_2_, 7 mM sodium acetate, and 247 mM mannitol binding to protoplasts was exclusively lateral. No UPP could be detected on bound or planktonic bacteria in either medium suggesting that it was not made under these conditions (Matthysse, manuscript in preparation). The factor determining whether UPP was produced appeared to be the calcium ion concentration. MS medium contains 3 mM CaCl_2_ at a pH of 5.6. Addition of calcium to media in which UPP is ordinarily synthesized resulted in reduced or undetectable UPP production by the bacteria and reduced bacterial binding (**Figure 2**).

In the absence of the production of UPP or cellulose bacterial binding appears to be reversible and the bacteria can be removed from the plant surface by water washing (Lippincott and Lippincott, 1967; Sykes and Matthysse, 1986). Cellulose production and irreversible bacterial binding appear to occur about 2–4 h after the inoculation of the bacteria into wound sites or plant cell suspension cultures (Lippincott and Lippincott, 1967; Matthysse, 1983; Neff and Binns, 1985). These experiments were all carried out in media which contained more than 3 mM calcium and thus there was probably little UPP produced by the bacteria.

In bacteria incubated with plant protoplasts in 60 mM CaCl_2_, 7 mM sodium acetate, and 247 mM mannitol pH 5.8 bacterial binding to the plant cells was observed to be lateral. Under these conditions, the T pilus was also localized laterally in the bacteria (Aguilar et al., 2011). However, when bacteria were grown under inducing conditions with low calcium ions, the T pilus was reported to be exclusively localized at the end of the bacteria (polar localization; Lai et al., 2000). Polar localization of the VirB proteins (except VirB2) which assemble the T-pilus in cells incubated under inducing conditions in low calcium concentrations was shown by Judd et al. (2005). The observations showing polar and lateral localization of the Ti pilus differ in the medium used which may affect the position of the pilus. Low calcium would favor the elaboration of the UPP which could conceivably help to direct the T pilus to the cell pole. Lateral vs polar attachment of the bacteria may also be affected by the plant surface to which the bacteria are attached. The experiment showing lateral orientation of the bacteria involved bacterial attachment to tobacco protoplasts. The receptors to which the bacteria bind are likely to differ in nature and/or orientation between intact plant cells and protoplasts. Thus, the lateral bacterial attachment observed by Aguilar et al. (2011) could be a result of using tobacco protoplasts. However, bacteria bound to glutaraldehyde-fixed carrot protoplasts were observed in both lateral and polar orientations (Matthysse et al., 1982).

The role of pTi in bacterial attachment is unclear. Genes on pTi which may be involved in binding have not been identified. It is possible that binding is mediated by the T pilus itself, in which case VirB2 which makes up the shaft of the pilus or VirB5 which is found at the tip of the pilus are the obvious candidates for the proteins involved (Aly and Baron, 2007; Christie et al., 2014). Some mutations in *virB5* which alter or delete the carboxy-terminal amino acids of the protein result in bacteria which can transfer pTi to other bacteria but when inoculated onto plants (*K. daigremontiana*) the bacteria were avirulent (Aly and Baron, 2007). It is not known which steps in DNA transfer are blocked in these mutants. Exogenous VirB5 enhanced DNA transfer from the bacteria to the plant as measured by a transient gene expression assay. The exogenous VirB5 had no effect on bacterial binding to roots under conditions where the majority of the binding is mediated by UPP (Lacroix and Citovsky, 2011). Whether VirB5 affects binding under conditions where UPP-mediated attachment is not seen is unknown. Binding of VirB2 to host proteins found on the surface of *Arabidopsis thaliana* roots has been described suggesting that VirB2 may play a role in bacterial attachment to host plants (Hwang and Gelvin, 2004). Thus VirB2 and VirB5 pilus proteins may play a role in bacterial attachment. Other genes which play a role in bacterial binding may also be located on pTi; these could potentially include both genes for adhesins or regulatory genes which control the expression of adhesin genes located elsewhere in the genome.

## CELLULOSE-MEDIATED ATTACHMENT

*Agrobacterium tumefaciens* like many other bacteria is capable of making cellulose (Matthysse et al., 1981). The genes required are located in two adjacent operons on the linear chromosome (Matthysse et al., 1995). The cellulose synthase CelA of agrobacteria shares a high degree of homology with the cellulose synthases of other proteobacteria including rhizobia, *Gluconacetobacter xylinus*, and *Escherichia coli*. *A. tumefaciens* and the rhizobia which produce cellulose synthesize the exopolysaccharide in microfibrils emerging from many points scattered over the cell surface. In contrast, in bacteria such as *G. xylinus* and *P. fluorescens* cellulose fibrils emerge from a linear array of sites on one side of the cell and the cellulose produced forms a sheet (Brown et al., 1976; Cannon and Anderson, 1991; Spiers et al., 2003). This difference in the geometry of cellulose production influences the type of aggregates the bacteria form in solution and on surfaces and correlates with sequence differences in the *celB* gene. Cellulose fibrils bind tightly to other cellulose fibrils and thus cellulose synthesis results in the formation of bacterial aggregates which may be free in solution or bound to the cellulose on the plant surface. Bacteria in aggregates of *A. tumefaciens* produced by cellulose tend to be tangled in the cellulose in random orientations. Cellulose-producing *A. tumefaciens* will also bind to non-living materials containing cellulose such as Whatman filter paper (Matthysse, 1983). The production of cellulose by attached bacteria results in the formation of large clumps of attached bacteria on filter paper as well as on plant surfaces.

Cellulose synthesis is known to be regulated by a number of genes. Mutations in c*elG* (*Atu8186*, the last gene in the operon containing *celABCG*) result in overproduction of cellulose (Matthysse et al., 2005). An RNA or protein product of the gene must be involved as the cellulose overproduction in a *celG* mutant can be reduced to wild-type levels by the provision of the gene on a plasmid. Mutations in *celI* (*Atu3105*) which has homology to transcriptional regulators also cause overproduction of cellulose (Matthysse et al., 2005). No additional information is available about the function of this gene.

In many bacteria including *A. tumefaciens* cellulose synthase (the product of the *celA* gene) can be directly regulated by c-di-GMP which binds to a *pilZ* site in the carboxy-terminal end of the protein (Amikam and Benziman, 1989; Ross et al., 1991). The active site where UDP-glucose is bound is located in the amino-terminal end. Regulation by c-di-GMP acts directly on the enzymatic activity of the protein and can be observed in cell-free extracts of the bacteria by measuring rate of incorporation of UDP-glucose into cellulose. Overexpression of either of two genes encoding a diguanylate cyclase, *Atu1297* or *Atu1060*, causes increased cellulose synthesis. A deletion of *Atu1297* (also known as *celR* or *pleD*) reduces the synthesis of cellulose and as well as (an)other undefined exopolysaccharide(s). This deletion also increased polar attachment of *A. tumefaciens* to the plant surface and biofilm formation on glass due to an increase in the amount of UPP present (Barnhart et al., 2013, 2014). Thus, regulation by c-di-GMP serves to integrate the synthesis of cellulose and UPP. However, *Atu1297* and *Atu1060* have other effects on virulence in addition to their effects on cellulose and UPP synthesis. To examine the effects of these genes on processes other than cellulose synthesis, the effects of overexpressing either *Atu1297* or *Atu1060* were examined in a cellulose synthase (*celA*) deletion mutant. Overexpression of either gene resulted in reduced virulence (Barnhart et al., 2013). Deletion of cellulose synthase by itself has little effect on virulence but does render bacterial binding more fragile so that the bacteria can be removed by water washing (Matthysse, 1983). Overproduction of cellulose causes the formation of large aggregates of bacteria on surfaces but has little effect on virulence (Matthysse et al., 2005).

## THE ROLE OF OTHER EXOPOLYSACCHARIDES: CYCLIC-β-1,2-D-GLUCAN, SUCCINOGLYCAN, LIPOPOLYSACCHARIDE, AND CURDLAN

Bacterial mutants (*chvA* and *chvB*) which fail to synthesize the periplasmic polysaccharide cyclic-β-1,2-D-glucan were the first mutants shown to be defective in binding to plant cells (Douglas et al., 1982). Inability to synthesize this polysaccharide has pleiotropic effects including increased sensitivity to osmotic stress, overproduction of succinoglycan, and reduced motility (Douglas et al., 1985; Puvanesarajah et al., 1985). The effects of *chvB* mutations are temperature sensitive. The ability to bind to plants, motility, and virulence are all restored in c*hvB* mutants when incubation of the bacteria with the plants is carried out at temperatures below 16°C (Bash and Matthysse, 2002). Addition of cyclic-β-1,2-D-glucan to the solution has no effect on the attachment of wild-type *A. tumefaciens* to plant cell surfaces (Puvanesarajah et al., 1985). It seems likely that the effect of *chvA* and *chvB* mutations is indirect, resulting from multiple defects caused by the absence of the glucan polysaccharide from the periplasmic space rather than from the absence of a molecule which plays a direct role in attachment.

Succinoglycan is the most abundant of the exopolysaccharides produced by *A. tumefaciens* growing on agar plates in the laboratory. However, its role in the life of the bacteria in nature remains obscure. Bacterial mutants unable to synthesize succinoglycan retain virulence and show no obvious defects in binding to plant surfaces (Tomlinson et al., 2010). Overproduction of succinoglycan is seen in *chvA, chvB*, and *exoR* mutants (Puvanesarajah et al., 1985; Tomlinson et al., 2010). All of these mutants show reduced binding to roots and reduced motility. However, unlike *chvA* and *chvB* mutants, *exoR* mutants retain virulence on potato disks. An *exoAexoR* double mutant that cannot make succinoglycan recovered the ability to bind to roots, but did not recover wild-type motility suggesting that the overproduction of succinoglycan was responsible for the lack of binding of the *exoR* mutants (Tomlinson et al., 2010). The role, if any, played by excess succinoglycan in the phenotype of *chvA* and *chvB* mutants is unknown.

There is little information about a possible role for LPS in the attachment of *A. tumefaciens* to plant cells. The addition of purified LPS from *A. tumefaciens* strain C58 inhibited bacterial binding to carrot suspension cells in MS medium (Whatley et al., 1976; Matthysse, 1987b). However, the effects of added bacterial substances on binding may be due to their ability to elicit plant defense reactions rather than a direct effect on binding. A study using an inhibitor of LPS biosynthesis found no effect on the initial attachment although the drug did inhibit the formation of cellulose fibrils (Goldman et al., 1992).

*Agrobacterium tumefaciens* strain C58 has intact genes for the biosynthesis of curdlan; however, this strain has not been observed to make curdlan. Curdlan synthase (*crdS*) mutants retain virulence and are able to colonize roots (Matthysse, unpublished observation). Other strains of *Agrobacterium* such as LTU50 and ATCC1379 are used in industry to produce large amounts of curdlan (McIntosh et al., 2005). These strains lack pTi and thus are not virulent. LTU50 is able to colonize plant roots and has been observed to bind to root hairs (Aracic et al., unpublished observations). Curdlan production in LTU50 is negatively regulated by the presence of combined nitrogen (McIntosh et al., 2005). When bacterial growth is limited by the absence of available nitrogen and an abundant carbon source such as glucose is present, the bacteria produce large amounts of curdlan. Bacteria growing in 4% glucose can convert 95% of this glucose into curdlan (McIntosh et al., 2005). LTU50 incubated with tomato roots in MS medium rapidly run out of combined nitrogen and begin to make curdlan. The bacteria embedded in a curdlan matrix form a blanket-like structure covering the roots. This structure is fragile and easily removed by water washing (Matthysse, unpublished observation). Bacteria embedded in curdlan are protected from phagocytosis by protists such as *Dictyostelium discoideum* (Aracic et al., unpublished observations). Thus, curdlan production is likely to increase bacterial survival in soil.

## PROTEIN ADHESINS

A 14-kDa calcium-binding protein named rhicadhesin has been reported to be involved in the binding of rhizobia and *A. tumefaciens* to root hairs (Smit et al., 1989a, b). Rhicadhesin is reported to be released from the surface of the bacterial cell when the cells are placed in medium with low concentrations of calcium. Addition of the purified protein inhibited the binding of rhizobia and *A. tumefaciens* to pea roots (Smit et al., 1992). The purified protein was also able to restore the binding of an *A. tumefaciens chvB* mutant to pea roots and virulence on *K. daigremontiana* (Swart et al., 1994). The gene encoding this protein has not been identified. However, the protein is made by rhizobia lacking the sym plasmid and by *A. tumefaciens* lacking pTi suggesting that the relevant gene(s) are chromosomal (Smit et al., 1987). There are many possible reasons why the rhicadhesin gene has not been identified. Among the likeliest is the existence of multiple copies of the gene so that a mutation in one copy has no evident phenotype or the possibility that mutations in the gene are lethal. The role of rhicadhesin in attachment remains uncertain. It was defined by its ability to inhibit bacterial attachment. The major case in which it promotes attachment involves its addition to *chvB* mutants. However, as discussed above the phenotype of these mutants probably result from indirect effects of the lack of cyclic-β-1,2-D-glucan. Thus, the mechanism of the restoration of the wild-type phenotype may be indirect. The experimental data do support a role for rhicadhesin in the structure and stability of the bacterial surface. The definition of its role in attachment will have to await the identification of the gene(s) encoding this protein.

Other protein adhesins which play a role in the binding of *R. leguminosarum* to roots have been identified. These include the Rap proteins which are secreted bacterial proteins that bind to the surface of the bacteria. RapA1 is a calcium-binding protein with two binding sites which agglutinates the bacteria by binding at the pole. These genes for these proteins are restricted to only a few members of the *Rhizobiaceae* (Ausmees et al., 2001). The overexpression of RapA1 from the gene cloned into a plasmid resulted in increased bacterial binding to roots but had no effect on binding to abiotic surfaces (Mongiardini et al., 2008). The gene is not required for nodulation. The suggested role for this protein is in root colonization by the bacteria. RapA2 is also a calcium-binding protein. It interacts with the acidic exopolysaccharide of the bacteria and is apparently a calcium-dependent lectin (Abdian et al., 2013). No genes homologous to the *rap* genes have been identified in *A. tumefaciens*.

Several genes on the cryptic plasmid pAT (*att* genes) have been identified as being involved in attachment. Transposon insertions in these genes block attachment in calcium-containing medium in which the UPP is not made (Matthysse, 1987a; Matthysse et al., 2000). The mutations have no effect on attachment in medium in which the UPP mediates the majority of bacterial attachment. Their effect on the synthesis of the T pilus is unknown. The genes cannot be required for virulence as bacterial strains lacking pAT are virulent (Nair et al., 2003). The transposon insertions in some of the *att* genes (*attC* and *attG*) resulted in dominant-negative mutations (Matthysse et al., 2008) suggesting that they act by causing the synthesis of partial proteins (affected gene translated to the site of the insertion) or partial protein complexes (only some of the genes in an operon expressed) perturbing the bacterial surface so as to block the ability of the bacteria to bind to plants in medium containing moderate levels of calcium ions. Whatever the mechanism of action of the transposon insertion mutations in genes found on pAT, it appears certain that the effects of these mutations, similar to those of the *chvA* and *chvB* mutations, on bacterial attachment are indirect and that the genes do not encode molecules directly involved in bacterial attachment.

## GENERAL CONCLUSIONS

**FIGURE 3:**
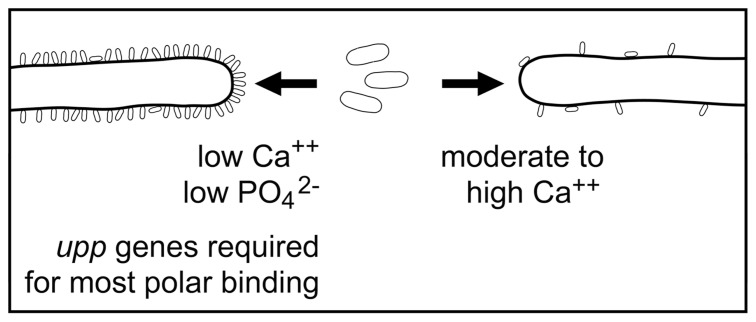
**A model of the initial binding of *A. tumefaciens* to root hairs**. In the presence of low levels of calcium UPP is formed and mediates polar bacterial attachment to the root hair surface. This results in binding of large numbers of bacteria to the root hair. The genes for synthesizing the UPP are required for this binding. In the presence of moderate or high levels of calcium UPP is not formed and bacterial binding is sparse.

It appears that *A. tumefaciens* has at least two mechanisms by which it can bind to plant surfaces (**Figure 3**). One, the UPP, is quite non-specific and aids the bacteria in binding to a wide variety of both animate and inanimate surfaces. This binding is visually striking because it is a polar attachment and results in the binding of large numbers of bacteria to the surface. The UPP is produced optimally under conditions of low calcium, low phosphate, and acidic pH. UPP-mediated binding to surfaces is likely to play a prominent role both in attachment to soil particles and in colonization of plant surfaces. The genes for the production of this exopolysaccharide are located on the chromosome and appear to be widely distributed in the agrobacteria and rhizobia.

The second mechanism of attachment is mediated by unknown molecule(s). It can be detected when the interactions between the bacteria and surfaces are carried out in media containing moderate to high concentrations of calcium where the UPP is not produced or by the examination of the binding of UPP mutants. The numbers of bacteria bound are very small when compared with bacterial binding mediated by the UPP. This UPP-independent attachment may result in both polar and lateral attachment to plant surfaces. It is not known what conditions control the polar vs lateral orientation of the bacterium or whether bacteria bound in these two orientations use different mechanisms of attachment. No mutants unable to show UPP-independent attachment have been identified. Thus it is not known whether more than one type of UPP-independent attachment exists nor is there any information on the genes or adhesins involved in this binding. It seems clear that the major mechanism of attachment of *A. tumefaciens* to surfaces both biological and inanimate has been identified as the binding of the UPP but there clearly remains more to be discovered about the surface interactions of this bacterium with its plant hosts particularly those which result in T-DNA transfer.

## Conflict of Interest Statement

The author declares that the research was conducted in the absence of any commercial or financial relationships that could be construed as a potential conflict of interest.

## References

[B1] Abdian P. L. Caramelo J. J. Ausmees N. Zorreguieta A. (2013). RapA2 is a calcium-binding lectin composed of two highly conserved cadherin-like domains that specifically recognize *Rhizobium leguminosarum* acidic exopolysaccharides. *J. Biol. Chem.* 288 2893–2904 10.1074/jbc.M112.411769 23235153 PMC3554953

[B2] Aguilar J. Cameron T. A. Zupan J. Zambryski P. (2011). Membrane and core periplasmic *Agrobacterium tumefaciens* virulence Type IV secretion system components localize to multiple sites around the bacterial perimeter during lateral attachment to plant cells. *MBio* 2:e00218–11 10.1128/mBio.00218-11 PMC3202754 22027007

[B3] Aly K. A. Baron C. (2007). The VirB5 protein localizes to the T-pilus tips in *Agrobacterium tumefaciens*. *Microbiology* 153 3766–3775 10.1099/mic.0.2007/010462-0 17975085

[B4] Amikam D. Benziman M. (1989). Cyclic diguanylic acid and cellulose synthesis in *Agrobacterium tumefaciens*. *J. Bacteriol.* 171 6649–6655 2556370 10.1128/jb.171.12.6649-6655.1989 PMC210559

[B5] Ausmees N. Jacobsson K. Lindberg M. (2001). A unipolarly located, cell-surface-associated agglutinin, RapA, belongs to a family of Rhizobium-adhering proteins (Rap) in *Rhizobium leguminosarum bv*. *trifolii. Microbiology* 147 549–559 10.1099/00221287-147-3-549 11238962

[B6] Barnhart D. M. Su S. Baccaro B. E. Banta L. M. Farrand S. K. (2013). CelR, an ortholog of the diguanylate cyclase PleD of Caulobacter, regulates cellulose synthesis in *Agrobacterium tumefaciens*. *Appl. Environ. Microbiol.* 79 7188–7202 10.1128/AEM.02148-13 24038703 PMC3837745

[B7] Barnhart D. M. Su S. Farrand S. K. (2014). A signaling pathway involving the diguanylate cyclase CelR and the response regulator DivK controls cellulose synthesis in *Agrobacterium tumefaciens*. *J. Bacteriol.* 196 1257–1274 10.1128/JB.01446-13 24443526 PMC3957726

[B8] Bash R. Matthysse A. G. (2002). Attachment to roots and virulence of a chvB mutant of *Agrobacterium tumefaciens* are temperature sensitive. *Mol. Plant Microbe Interact.* 15 160–163 10.1094/MPMI.2002.15.2.160 11876426

[B9] Brown R. M. Jr. Willison J. H. M. Richardson C. L. (1976). Cellulose biosynthesis in *Acetobacter xylinum*: visualization of the site of synthesis and direct measurement of the in vivo process. *Proc. Natl. Acad. Sci. U.S.A.* 73 4565–4569 10.1073/pnas.73.12.4565 1070005 PMC431544

[B10] Bundock P. den Dulk-Ras A. Beijersbergen A. Hooykaas P. J. (1995). Trans-kingdom T-DNA transfer from *Agrobacterium tumefaciens* to *Saccharomyces cerevisiae*. *EMBO J.* 14 3206–3214 7621833 10.1002/j.1460-2075.1995.tb07323.x PMC394382

[B11] Cannon R. E. Anderson S. M. (1991). Biogenesis of bacterial cellulose. *Crit. Rev. Microbiol.* 17 435–447 10.3109/10408419109115207 2039586

[B12] Christie P. J. Whitaker N. Gonzalez-Rivera C. (2014). Mechanism and structure of the bacterial type IV secretion systems. *Biochim. Biophys. Acta*. 10.1016/j.bbamcr.2013.12.019 [Epub ahead of print]. PMC4061277 24389247

[B13] Danhorn T. Fuqua C. (2007). Biofilm formation by plant-associated bacteria. *Annu. Rev. Microbiol.* 61 401–422 10.1146/annurev.micro.61.080706.093316 17506679

[B14] Douglas C. J. Halperin W. Nester E. W. (1982). *Agrobacterium tumefaciens* mutants affected in attachment to plant cells. *J. Bacteriol.* 152 1265–1275 6292165 10.1128/jb.152.3.1265-1275.1982 PMC221638

[B15] Douglas C. J. Staneloni R. J. Rubin R. A. Nester E. W. (1985). Identification and genetic analysis of an *Agrobacterium tumefaciens* chromosomal virulence region. *J. Bacteriol.* 161 850–860 2982791 10.1128/jb.161.3.850-860.1985 PMC214975

[B16] Downie J. A. (2010). The roles of extracellular proteins, polysaccharides and signals in the interactions of rhizobia with legume roots. *FEMS Microbiol. Rev.* 34 150–170 10.1111/j.1574-6976.2009.00205.x 20070373

[B17] Goldman R. C. Capobianco J. O. Doran C. C. Matthysse A. G. (1992). Inhibition of lipopolysaccharide synthesis in *Agrobacterium tumefaciens* and *Aeromonas salmonicida*. *J. Gen. Microbiol.* 138 (Pt 7) 1527–1533 10.1099/00221287-138-7-1527 1324975

[B18] Gurlitz R. H. Lamb P. W. Matthysse A. G. (1987). Involvement of carrot cell surface proteins in attachment of *Agrobacterium tumefaciens*. *Plant Physiol*. 83 564–568 10.1104/pp.83.3.564 16665289 PMC1056405

[B19] Hwang H. H. Gelvin S. B. (2004). Plant proteins that interact with VirB2, the *Agrobacterium tumefaciens* pilin protein, mediate plant transformation. *Plant Cell* 16 3148–3167 10.1105/tpc.104.026476 15494553 PMC527204

[B20] Jeter C. Matthysse A. G. (2005). Characterization of the binding of diarrheagenic strains of *E. coli* to plant surfaces and the role of curli in the interaction of the bacteria with alfalfa sprouts. *Mol. Plant Microbe Interact.* 18 1235–1242 10.1094/MPMI-18-1235 16353558

[B21] Judd P. K. Kumar R. B. Das A. (2005). Spatial location and requirements for the assembly of the *Agrobacterium tumefaciens* type IV secretion apparatus. *Proc. Natl. Acad. Sci. U.S.A*. 102 11498–11503 10.1073/pnas.0505290102 16076948 PMC1183602

[B22] Lacroix B. Citovsky V. (2011). Extracellular VirB5 enhances T-DNA transfer from *Agrobacterium* to the host plant. *PLoS ONE* 6:e25578 10.1371/journal.pone.0025578 PMC3196495 22028781

[B23] Lai E.-M. Chesnokova O. Banta L. M. Kado C. I. (2000). Genetic and Environmental factors affecting T-pilin export and T-pilus biogenesis in relation to flagellation of affecting of *Agrobacterium tumefaciens*. *J. Bacteriol.* 182 3705–3716 10.1128/JB.182.13.3705-3716.2000 10850985 PMC94541

[B24] Laus M. C. Logman T. J. Lamers G. E. Van Brussel A. A. Carlson R. W. Kijne J. W. (2006). A novel polar surface polysaccharide from *Rhizobium leguminosarum* binds host plant lectin. *Mol. Microbiol.* 59 1704–1713 10.1111/j.1365-2958.2006.05057.x 16553877

[B25] Li G. Brown P. J. Tang J. X. Xu J. Quardokus E. M. Fuqua C. (2012). Surface contact stimulates the just-in-time deployment of bacterial adhesins. *Mol. Microbiol.* 83 41–51 10.1111/j.1365-2958.2011.07909.x 22053824 PMC3245333

[B26] Lippincott B. B. Lippincott J. A. (1969). Bacterial attachment to a specific wound site as an essential stage in tumor initiation by *Agrobacterium tumefaciens*. *J. Bacteriol.* 97 620–628 5773014 10.1128/jb.97.2.620-628.1969 PMC249736

[B27] Lippincott B. B. Whatley M. H. Lippincott J. A. (1977). Tumor induction by *Agrobacterium* involves attachment of bacterium to A site on host plant-cell wall. *Plant Physiol.* 59 388–390 10.1104/pp.59.3.388 16659858 PMC542409

[B28] Lippincott J. A. Lippincott B. B. (1967). Time required for tumour initiation by *Agrobacterium tumefaciens* on pinto bean leaves. *Nature* 213 596–598 10.1038/213596b0 6032253

[B29] Lippincott J. A. Lippincott B. B. (1975). The genus *Agrobacterium* and plant tumorigenesis. *Annu. Rev. Microbiol.* 29 377–405 10.1146/annurev.mi.29.100175.002113 1180518

[B30] Lugtenberg B. J. Kravchenko L. V. Simons M. (1999). Tomato seed and root exudate sugars: composition, utilization by Pseudomonas biocontrol strains and role in rhizosphere colonization. *Environ. Microbiol.* 1 439–446 10.1046/j.1462-2920.1999.00054.x 11207764

[B31] Matthysse A. G. (1983). Role of bacterial cellulose fibrils in *Agrobacterium tumefaciens* infection. *J. Bacteriol.* 154 906–915 6302086 10.1128/jb.154.2.906-915.1983 PMC217544

[B32] Matthysse A. G. (1986). Initial interactions of *Agrobacterium tumefaciens* with plant host cells. *Crit. Rev. Microbiol.* 13 281–307 10.3109/10408418609108740 3533427

[B33] Matthysse A. G. (1987a). Characterization of nonattaching mutants of *Agrobacterium tumefaciens*. *J. Bacteriol.* 169 313–323 3025176 10.1128/jb.169.1.313-323.1987 PMC211770

[B34] Matthysse A. G. (1987b). Effect of plasmid pSa and of auxin on attachment of *Agrobacterium tumefaciens* to carrot cells. *Appl. Environ. Microbiol.* 53 2574–2582 16347473 10.1128/aem.53.10.2574-2582.1987 PMC204148

[B35] Matthysse A. G. (1996). “Adhesion in the rhizosphere,” in *Molecular and Ecological Diversity of Bacterial Adhesion* edsFletcher M. Savage D. (New York, NY: John Wiley & Sons, Inc.) 129–153

[B36] Matthysse A. G. Holmes K. Gurlitz R. H. (1982). Binding of *Agrobacterium tumefaciens* to carrot protoplasts. *Physiol. Plant Pathol.* 20 27–33 10.1016/0048-4059(82)90020-0

[B37] Matthysse A. G. Holmes K. V. Gurlitz R. H. (1981). Elaboration of cellulose fibrils by *Agrobacterium tumefaciens* during attachment to carrot cells. *J. Bacteriol.* 145 583–595 7462151 10.1128/jb.145.1.583-595.1981 PMC217308

[B38] Matthysse A. G. Jaeckel P. Jeter C. (2008). attG and attC mutations of *Agrobacterium tumefaciens* are dominant negative mutations that block attachment and virulence. *Can. J. Microbiol.* 54 241–247 10.1139/W08-005 18388996

[B39] Matthysse A. G. Marry M. Krall L. Kaye M. Ramey B. E. Fuqua C. (2005). The effect of cellulose overproduction on binding and biofilm formation on roots by *Agrobacterium tumefaciens*. *Mol. Plant Microbe Interact.* 18 1002–1010 10.1094/MPMI-18-1002 16167770

[B40] Matthysse A. G. White S. Lightfoot R. (1995). Genes required for cellulose synthesis in *Agrobacterium tumefaciens*. *J. Bacteriol.* 177 1069–1075 7860585 10.1128/jb.177.4.1069-1075.1995 PMC176703

[B41] Matthysse A. G. Wyman P. M. Holmes K. V. (1978). Plasmid-dependent attachment of *Agrobacterium tumefaciens* to plant tissue culture cells. *Infect. Immun.* 22 516–522 730370 10.1128/iai.22.2.516-522.1978 PMC422186

[B42] Matthysse A. G. Yarnall H. Boles S. B. McMahan S. (2000). A region of the *Agrobacterium tumefaciens* chromosome containing genes required for virulence and attachment to host cells. *Biochim. Biophys. Acta* 1490 208–212 10.1016/S0167-4781(99)00250-X 10786639

[B43] McIntosh M. Stone B. A. Stanisich V. A. (2005). Curdlan and other bacterial (1→3)-beta-D-glucans. *Appl. Microbiol. Biotechnol.* 68 163–173 10.1007/s00253-005-1959-5 15818477

[B44] Mongiardini E. J. Ausmees N. Perez-Gimenez J. Julia A. M. Ignacio Q. J. Lopez-Garcia S. L. (2008). The rhizobial adhesion protein RapA1 is involved in adsorption of rhizobia to plant roots but not in nodulation. *FEMS Microbiol. Ecol.* 65 279–288 10.1111/j.1574-6941.2008.00467.x 18393991

[B45] Nair G. R. Liu Z. Binns A. N. (2003). Reexamining the role of the accessory plasmid pAtC58 in the virulence of *Agrobacterium tumefaciens* strain C58. *Plant Physiol.* 133 989–999 10.1104/pp.103.030262 14551325 PMC281596

[B46] Neff N. T. Binns A. N. (1985). *Agrobacterium tumefaciens* interaction with suspension-cultured tomato cells. *Plant Physiol.* 77 35–42 10.1104/pp.77.1.35 16664024 PMC1064452

[B47] Neff N. T. Binns A. N. Brandt C. (1987). Inhibitory effects of a pectin-enriched tomato cell wall fraction on *Agrobacterium tumefaciens* binding and tumor formation. *Plant Physiol.* 83 525–528 10.1104/pp.83.3.525 16665282 PMC1056398

[B48] Piers K. L. Heath J. D. Liang X. Stephens K. M. Nester E. W. (1996). *Agrobacterium tumefaciens*-mediated transformation of yeast. *Proc. Natl. Acad. Sci. U.S.A.* 93 1613–1618 10.1073/pnas.93.4.1613 8643679 PMC39990

[B49] Puvanesarajah V. Schell F. M. Stacey G. Douglas C. J. Nester E. W. (1985). Role for 2-linked-beta-D-glucan in the virulence of *Agrobacterium tumefaciens*. *J. Bacteriol.* 164 102–106 4044517 10.1128/jb.164.1.102-106.1985 PMC214216

[B50] Ramey B. E. Matthysse A. G. Fuqua C. (2004). The FNR-type transcriptional regulator SinR controls maturation of *Agrobacterium tumefaciens* biofilms. *Mol. Microbiol.* 52 1495–1511 10.1111/j.1365-2958.2004.04079.x 15165250

[B51] Ramirez C. Alexander M. (1980). Evidence suggesting protozoan predation on rhizobium associated with germinating seeds and in the rhizosphere of beans (*Phaseolus vulgaris* L.). *Appl. Environ. Microbiol.* 40 492–499 16345628 10.1128/aem.40.3.492-499.1980 PMC291611

[B52] Ross P. Mayer R. Benziman M. (1991). Cellulose biosynthesis and function in bacteria. *Microbiol. Rev.* 55 35–58 2030672 10.1128/mr.55.1.35-58.1991 PMC372800

[B53] Smit G. Kijne J. W. Lugtenberg B. J. (1987). Involvement of both cellulose fibrils and a Ca^2^^+^-dependent adhesin in the attachment of *Rhizobium leguminosarum* to pea root hair tips. *J. Bacteriol.* 169 4294–4301 3624205 10.1128/jb.169.9.4294-4301.1987 PMC213743

[B54] Smit G. Kijne J. W. Lugtenberg B. J. (1989a). Roles of flagella, lipopolysaccharide, and a Ca^2^^+^-dependent cell surface protein in attachment of *Rhizobium leguminosarum* biovar viciae to pea root hair tips. *J. Bacteriol.* 171 569–572 2914856 10.1128/jb.171.1.569-572.1989 PMC209624

[B55] Smit G. Logman T. J. Boerrigter M. E. Kijne J. W. Lugtenberg B. J. (1989b). Purification and partial characterization of the Rhizobium leguminosarum biovar viciae Ca^2^^+^-dependent adhesin, which mediates the first step in attachment of cells of the family Rhizobiaceae to plant root hair tips. *J. Bacteriol.* 171 4054–4062 2738027 10.1128/jb.171.7.4054-4062.1989 PMC210161

[B56] Smit G. Swart S. Lugtenberg B. J. Kijne J. W. (1992). Molecular mechanisms of attachment of Rhizobium bacteria to plant roots. *Mol. Microbiol.* 6 2897–2903 10.1111/j.1365-2958.1992.tb01748.x 1479881

[B57] Spiers A. J. Bohannon J. Gehrig S. M. Rainey P. B. (2003). Biofilm formation at the air-liquid interface by the *Pseudomonas fluorescens* SBW25 wrinkly spreader requires an acetylated form of cellulose. *Mol. Microbiol.* 50 15–27 10.1046/j.1365-2958.2003.03670.x 14507360

[B58] Stewart P. S. Costerton J. W. (2001). Antibiotic resistance of bacteria in biofilms. *Lancet* 358 135–138 10.1016/S0140-6736(01)05321-1 11463434

[B59] Swart S. Lugtenberg B. Smit G. Kijne J. W. (1994). Rhicadhesin-mediated attachment and virulence of an *Agrobacterium tumefaciens* chvB mutant can be restored by growth in a highly osmotic medium. *J. Bacteriol.* 176 3816–3819 8206861 10.1128/jb.176.12.3816-3819.1994 PMC205572

[B60] Sykes L. C. Matthysse A. G. (1986). Time required for tumor induction by *Agrobacterium tumefaciens*. *Appl. Environ. Microbiol.* 52 597–598 3767365 10.1128/aem.52.3.597-598.1986 PMC203583

[B61] Tomlinson A. D. Fuqua C. (2009). Mechanisms and regulation of polar surface attachment in *Agrobacterium tumefaciens*. *Curr. Opin. Microbiol.* 12 708–714 10.1016/j.mib.2009.09.014 19879182 PMC2783196

[B62] Tomlinson A. D. Ramey-Hartung B. Day T. W. Merritt P. M. Fuqua C. (2010). *Agrobacterium tumefaciens* ExoR represses succinoglycan biosynthesis and is required for biofilm formation and motility. *Microbiology* 156 2670–2681 10.1099/mic.0.039032-0 20576688 PMC3068688

[B63] Wagner V. T. Matthysse A. G. (1992). Involvement of a vitronectin-like protein in attachment of *Agrobacterium tumefaciens* to carrot suspension culture cells. *J. Bacteriol.* 174 5999–6003 1381711 10.1128/jb.174.18.5999-6003.1992 PMC207141

[B64] Whatley M. H. Bodwin J. S. Lippincott B. B. Lippincott J. A. (1976). Role of *Agrobacterium* cell envelope lipopolysaccharide in infection site attachment. *Infect. Immun.* 13 1080–1083 1278998 10.1128/iai.13.4.1080-1083.1976 PMC420720

[B65] Williams A. Wilkinson A. Krehenbrink M. Russo D. M. Zorreguieta A. Downie J. A. (2008). Glucomannan-mediated attachment of *Rhizobium leguminosarum* to pea root hairs is required for competitive nodule infection. *J. Bacteriol.* 190 4706–4715 10.1128/JB.01694-07 18441060 PMC2446804

[B66] Xu J. Kim J. Danhorn T. Merritt P. M. Fuqua C. (2012). Phosphorus limitation increases attachment in *Agrobacterium tumefaciens* and reveals a conditional functional redundancy in adhesin biosynthesis. *Res. Microbiol.* 163 674–684 10.1016/j.resmic.2012.10.013 23103488 PMC3656598

[B67] Xu J. Kim J. Koestler B. J. Choi J. H. Waters C. M. Fuqua C. (2013). Genetic analysis of *Agrobacterium tumefaciens* unipolar polysaccharide production reveals complex integrated control of the motile-to-sessile switch. *Mol. Microbiol.* 89 929–948 10.1111/mmi.12321 23829710 PMC3799846

